# Governance Processes and Challenges for Reservation of Antimicrobials Exclusively for Human Use and Restriction of Antimicrobial Use in Animals

**DOI:** 10.1017/jme.2022.80

**Published:** 2022

**Authors:** J. Scott Weese, Guilherme Antonio Da Costa Junior, Bruno Gonzalez-Zorn, Laura Y. Hardefeldt, Jorge Matheu, Gerard Moulin, Stephen W. Page, Ruby Singh, Junxia Song, Olafur Valsson

**Affiliations:** 1: UNIVERSITY OF GUELPH, ONTARIO, CANADA; 2: MISSION OF BRAZIL TO THE EUROPEAN UNION, BRUSSELS, BELGIUM; 3: COMPLUTENSE UNIVERSITY, MADRID, SPAIN; 4: UNIVERSITY OF MELBOURNE, MELBOURNE, AUSTRALIA; 5: NATIONAL CENTRE FOR ANTIMICROBIAL STEWARDSHIP, AUSTRALIA; 6: WORLD HEALTH ORGANIZATION, GENEVA, SWITZERLAND; 7: FRENCH AGENCY FOR FOOD, ENVIRONMENTAL AND OCCUPATIONAL HEALTH & SAFETY (ANSES); 8: VETERINARY CLINICAL PHARMACOLOGY AND TOXICOLOGY, ADVANCED VETERINARY THERAPEUTICS IN NEWTOWN, AUSTRALIA; 9: UNIVERSITY OF SYDNEY, SYDNEY, AUSTRALIA; 10: FDA, ROCKVILLE, MARYLAND, USA; 11: FOOD AND AGRICULTURE ORGANIZATION OF UNITED NATIONS, ROME, ITALY; 12: WORLD ORGANISATION FOR ANIMAL HEALTH, PARIS, FRANCE

**Keywords:** Antimicrobial Resistance, Antimicrobial Stewardship, One Health, Agriculture

## Abstract

The majority of antimicrobials that are produced are administered to animals, particularly food animals. While the overall impact of antimicrobial use in animals on antimicrobial resistance in humans and the environment is unclear, it undeniably has a role. Yet, some degree of antimicrobial use in animals is necessary for animal health and welfare purposes. Balancing the benefits and risks of antimicrobial use in animals is challenging because of the complexity of the problem and limitations in available data. However, a range of measures can be implemented to reduce, refine and optimize antimicrobial use in animals, with a goal of minimizing the impact on human and environmental health while maintaining necessary therapeutic use in animals. A pandemic instrument can provide the necessary foundation for the whole-of-society and whole-of government One Health approach that is required to strengthen surveillance, communication, collaboration, and action.

## Introduction

The silent pandemic of antimicrobial resistance (AMR) is an important One Health issue as antimicrobials are used extensively in humans, animals, and on plants, and antimicrobial resistant bacteria can develop, disseminate, and have impacts on human, animal, and environmental health. Antimicrobials are used extensively in animals, particularly in food animal production, and most antimicrobials that are used in animals are from medically important drug classes that are used in humans.

The role of antimicrobial use (AMU) in animals on AMR in humans is poorly quantified. It is reasonable to assume that most AMR in humans results from AMU in humans, while most AMR in animals results from AMU in animals. However, AMR in some zoonotic pathogens can result in significant disease burdens in humans, which can vary by country, and AMU in animals undoubtedly contributes to some degree towards AMR in humans. A recent study estimating the burden of AMR on humans identified six leading pathogens associated with AMR deaths, some of which (most notably *Escherichia coli* and *Staphylococcus aureus*) can be associated with zoonotic infection.^1^ The impact of AMU in animals is most readily quantifiable when assessing the burden of AMR in foodborne pathogens. However, food is not the only potential source of exposure to zoonotic pathogens. Transmission can also result from direct contact with animals or their environments, as well as dissemination of AMR genes between bacteria. Environmentally acquired infections from bacteria of animal-origin and transmission of resistance determinants are poorly understood and require further study. While AMU in animals likely accounts for a minority of AMR issues in humans, given the massive and accelerating scope of global AMR, a minor contribution could still account for large numbers of total lives lost, disability-adjusted life years lost and high direct and indirect economic costs. Further, the ultimate destiny of antimicrobials is often the environment via feces, urine or disposal of antimicrobial-containing feed or water, with poorly understood environmental impacts.

Recognition of the potential impact of AMU in animals can lead to logical efforts to minimize AMU and the impact on AMR, with a parallel (and sometimes conflicting) goal of ensuring that antimicrobials are properly used, but only when necessary. Restriction is often the focus of discussion of potential regulatory approaches to AMU in animals. However, elimination of AMU in animals is not a realistic goal. As long as animals are kept for food, as companions, work, or conservation, there will be medical and ethical needs to treat them. Therefore, efforts need to be directed at reducing the need for antimicrobials, optimizing AMU in animals, maximizing the positive impacts while minimizing adverse effects on humans, animals, and the environment. The objective of this paper is to discuss potential governance approaches to optimizing AMU in animals within a pandemic instrument that uses a broad whole-of-society and whole-of government One Health approach, while highlighting the inherent and often under-appreciated complexities.

## Antimicrobial Use in Animals

Antimicrobial stewardship aims to optimize AMU, and an understanding of how antimicrobials are used in animals is required to develop and evaluate potential interventions. Antimicrobial use in animals can be divided into four categories, although the lines between them are sometimes indistinct (Table 1).

**Table 1:** Antimicrobial Use Definitions

Type of Use	Definition
Treatment	Administration of an antimicrobial agent to an individual or a group of animals showing clinical signs of an infectious disease.^2^
Prophylaxis/prevention of disease	Administration of an antimicrobial agent to an individual or a group of animals at risk of acquiring a specific infection or in a specific situation where infectious disease is likely to occur if the antimicrobial agent is not administered.^3^
Metaphylaxis/disease control	Administration of an antimicrobial agent to a group of animals containing sick animals and healthy animals (presumed to be infected), to minimize or resolve clinical signs and to prevent further spread of the disease.^4^
Growth promotion	Administration of antimicrobial agents to animals to increase the rate of weight gain or the efficiency of feed utilisation.^5^

AMU can also be separated into veterinary and non-veterinary medical use, categories that can also be indistinct. This categorization typically refers to the intended purpose of use (impacting disease vs solely impacting growth), with non-veterinary use being administration of antimicrobial agents for a purpose other than to treat, control or prevent infectious diseases (i.e., growth promotion). However, ‘veterinary’ uses can also be undertaken without involvement of veterinary professionals (with owner-sourced antimicrobials) or with limited direct veterinary involvement (e.g., owner-directed treatment using antimicrobials sourced from a veterinarian).

An additional factor when considering AMU in animals is the breadth of the term ‘animals’. Antimicrobials are used in diverse species, including terrestrial food animals (e.g., cattle, swine, goats, sheep, camels, poultry), aquatic food animals (e.g., finfish, crustaceans, amphibians), companion animals (e.g., dogs, cats), working animals (e.g., horses, donkeys, water buffalo, service animals, detection animals), performance animals (e.g. horses), fibre and fur bearing species (e.g., camelids, mink), bees and wildlife (including captive species that may be critically endangered). Even within those categories, there can be marked differences in AMU and AMR, with further major differences even within animal species (e.g., beef cattle vs. dairy cattle vs. veal calves). This highlights the challenges when addressing “AMU in animals” as “animals” represents a highly heterogenous group with different disease risks, AMU practices, AMR challenges and risks of zoonotic transmission of antimicrobial resistant organisms. A further consideration is marked differences in animal management within and between countries, especially between high income countries (HICs) and lower- and middle-income countries (LMICs).

Intended use of animals is yet another consideration. Economic factors may be a driving influence on management, prevention, diagnostic, and treatment considerations. Loss of animals or decreased production have economic impacts, potentially with downstream social consequences, especially for subsistence farmers and small stakeholders. Working equids may be critical to individual farmers. Companion animals may have profound emotional importance to individuals.

Administration practices also vary between species and management systems. This can include specific treatment of individual animals, treatment of a group of animals through direct dosing (e.g., administration of injectable antimicrobials to all individuals within a group of animals) and indirect group treatment through administration of antimicrobials in water or feed. In some situations, treatment of individual animals is difficult (e.g., large group of terrestrial food animals) or usually impossible (e.g., aquaculture).

## How Antimicrobials Are Accessed for Use in Animals

An understanding of how antimicrobials are accessed and used is fundamental to considerations of how to restrict or reserve drugs for human use. Access to antimicrobials is highly variable internationally, ranging from prescription-only to unrestricted over-the-counter access. Oversight can include animal-level prescription by a veterinarian, farm-level prescription, farm- or animal-level support from trained lay personnel (particularly in areas with limited access to veterinarians), recommendations from variably trained (and often untrained in animal disease) pharmacists, or no guidance whatsoever.

Dispensing mechanisms are similarly variable. They include direct sale from a veterinarian who has prescribed the drug (the main pathway in many regions), prescription from a veterinarian to be filled at a pharmacy, over-the-counter (non-prescription) purchase from a veterinary clinic, pharmacy or other source, purchase of antimicrobial-containing feed from a feed mill, purchase of compounded antimicrobials, purchase of antimicrobials through online sources and direct importation of active pharmaceutical ingredients. Numerous potential pathways for antimicrobial access add significant complexity to measures to monitor and control AMU in animals. While easier to obtain, the mass of antimicrobials that are purchased or prescribed do not necessarily accurately reflect the amount of product administered to animals by the end user. International efforts to facilitate and encourage (or require) collection of reliable and standardized (or at least comparable) AMU data are needed to better understand AMU patterns, to evaluate the impacts of AMU and to provide benchmarking data for interventions. Recognizing the challenges for some regions, these efforts should include collection of data at the farm and animal level, not simply overall production, importation, or sales data.

A short-term goal should be development of international (quadripartite) guidance for AMU in animals that considers risks posed by AMU in animals and the importance of the antimicrobial in humans and animals, along with examination of potential unintended consequences of restriction, to provide guidance for regulation and use of antimicrobials in animals. Ideally, this should involve separate considerations for food and companion animals because of different use patterns, human contact, zoonotic pathogens and human-animal bond considerations. A pandemic instrument can facilitate such an effort through efforts to promote, support, strengthen and sustain One Health related activities. It can further encourage and support harmonization of communication and guidance from members of the quadripartite that currently have separate, unlinked and sometimes conflicting approaches to AMU and AMR in animals.

## Antimicrobial Prioritization Efforts

Antimicrobial prioritization efforts have been undertaken by various groups and countries and can be the foundation of monitoring and restriction efforts. The World Health Organization’s Critically Important Antimicrobial list^6^ and the European Medicine’s Agency’s Categorization of Antimicrobials for Use in Animals^7^ are notable prioritization efforts. National lists have also been developed by some countries, typically HICs. Prioritization efforts are based on the importance of the drug in human medicine and the potential impact of AMU in animals on AMR in important human pathogens. Classifications of most drug classes tend to be similar between guidelines, but there are some notable differences. Some of these differences can be accounted for by regionally different antimicrobial access, AMU, AMR and disease patterns, or through application of different ranking criteria. Other documents refer to the importance of antimicrobial agents in human^8^ or veterinary^9^ medicine, but do not include assessment of the likelihood and consequences of AMR. Harmonization of these approaches is lacking. A short-term goal should be development of international (quadripartite) guidance for AMU in animals that considers risks posed by AMU in animals and the importance of the antimicrobial in humans and animals, along with examination of potential unintended consequences of restriction, to provide guidance for regulation and use of antimicrobials in animals. Ideally, this should involve separate considerations for food and companion animals because of different use patterns, human contact, zoonotic pathogens and human-animal bond considerations. A pandemic instrument can facilitate such an effort through efforts to promote, support, strengthen and sustain One Health related activities. It can further encourage and support harmonization of communication and guidance from members of the quadripartite that currently have separate, unlinked and sometimes conflicting approaches to AMU and AMR in animals.

## Possible Mechanisms to Regulate Antimicrobial use in Animals

Numerous potential approaches can be taken to regulating and restricting AMU in animals (Table 2). While it is reasonable to suspect that most or all of these would have beneficial impacts on AMU and correspondingly at least some reduction in AMR, data evaluating the impact of individual approaches are largely lacking and impacts likely vary between animal species, production system types and countries. Almost all directly focus on AMU, but measures to improve animal health systems must also be considered, as improved animal health systems should reduce illness in animals and the need for antimicrobials. While not addressing AMU directly, improved animal health systems (including biosecurity, vaccines and antimicrobial alternatives) could have the greatest impact on AMU, with parallel benefits in the economics of food production and animal welfare. A pandemic instrument can facilitate global implementation of animal health standards (Codex texts), help identify and address gaps in regulation of and access to antimicrobials and antimicrobial alternatives, mobilize adequate financing and strengthen supporting surveillance systems,

**Table 2:** Potential Regulatory Measures to Restrict, Reduce or Optimize Antimicrobial Access and Use in Animals

Mechanism	Action
Authorization (registration) of antimicrobial products	Banning selected use of specific antimicrobials in food animals or all animals
	Banning all use of specific antimicrobials in food animals or all animals
Antimicrobial drug access	Banning over-the-counter sale (sale without a veterinary prescription) of specific antimicrobials (e.g. medically important antimicrobials)
	Requiring a prescription for antimicrobial-containing feeds
	Restricting personal importation of active pharmaceutical ingredients
	Restricting personal importation of antimicrobial drug products
	Regulation of internet purchases of antimicrobials
Veterinary prescribing and dispensing	Restricting extra-label drug use
	Requiring inclusion of the indication for use on a prescription
	Regulation of profit margins on antimicrobials sold by veterinarians
	Regulating veterinarians’ prescribing powers
Antimicrobial use in food animals	Restricting use of antimicrobials for prophylaxis
	Banning the use of medically important antimicrobials for growth promotion
Education	Mandatory AMU and AMR continuing education and training for groups involved in AMU (e.g. veterinarians, producers)
Surveillance and benchmarking	AMU metric-based restrictions (e.g. Danish yellow card system)^10^
Animal health systems	Implementation and monitoring of animal health system standards (including antimicrobial stewardship programs)
Compliance & Enforcement	Compliance monitoring and enforcement of regulatory measures
Other	Regulation of compounding of antimicrobials
	Addressing problems with substandard, falsified or illicit antimicrobials
	Banning importation of animals/animal products treated with certain antimicrobials or growth promoters

## Potential Barriers to Regulation/Challenges to Regulation

While there is a large toolbox of options, there may be implementation challenges. Comprehensive discussion of the approaches in Table 2 is beyond the scope of this manuscript. Examples of issues are discussed below; however, it is important to note that no single approach is expected to have a profound effect. Rather a multimodal approach using a variety of tools will be required to have effective and sustained impacts.

Reducing antimicrobial prophylaxis and group treatments are important goals, yet this approach can be complicated. For example, recent EU legislation^11^ requires that antimicrobial prophylaxis should be limited to individual animals and only under specific conditions, and that prophylactic antimicrobials should not be administered “routinely” or to compensate for poor hygiene or management. Yet, “routinely” can be subjective, as can the ‘acceptable’ level of hygiene and management. The concept may be that antimicrobials should not be used as part of the standard production system, like vaccinations; however, it can be challenging to provide clear guidance that is effective at limiting AMU but does not adversely impact justifiable uses. This is addressed somewhat through allowing use in “exceptional cases” and to “an individual animal or restricted number of animals when the risk for infection is very high or its consequences are likely to be severe.” Adding subjectivity can be problematic, but is a practical necessity as exceptions are needed from animal health and welfare standpoints. While beneficial at the population level, prophylaxis prohibitions aimed at containing widespread unnecessary use in large groups can potentially have negative consequences for more targeted situations where the risk and implications of infections can be high (e.g., surgical prophylaxis) and use would be limited and short term. There are also issues with the blurred line between metaphylaxis/disease control (generally accepted when the risk is reasonable), prophylaxis (discouraged) and growth promotion (ideally ceased).

Antimicrobial authorization (registration) is a clear target for regulatory approaches. Antimicrobials are authorized for use in specific animal species and disease situations (e.g., treatment of cattle with respiratory disease). Extra-label drug use (ELDU) is the use of an antimicrobial that deviates from the product label and could include use of the drug in a different species, for a different indication, at a different dose or frequency, and use via a different route of administration. Restricting ELDU of an antimicrobial is a potential stewardship approach used by regulatory authorities; however, there are potential unintended consequences. Authorization tends to focus on common species and conditions, so there may be no approved options for certain diseases. For some minor species (e.g., small ruminants, rabbits), there may be few or no appropriate and authorized treatment options. Further, newer antimicrobials with the most specific label claims may be higher tier drugs (e.g., 3^rd^ generation cephalosporins, fluoroquinolones) and less desirable from a stewardship standpoint compared to older, narrow spectrum, lower tier options that may not be similarly authorized but would be effective. Thus, the concept of using authorized drugs whenever possible, as exemplified by the use of a cascade approach to drug selection, may be highly appropriate in most scenarios, but suboptimal in others. Restrictions in ELDU could therefore be useful in most scenarios but potentially harmful in others.

Control measures also must address equitable resource allocation as LMICs may face a greater burden from restrictions if they lack systems to counterbalance those effects (e.g., inability to rapidly improve management systems; less access to veterinary care, diagnostic testing, vaccines and antimicrobial alternatives; greater challenges implementing surveillance systems). Ideally, veterinarians would have at least some control over antimicrobials, such as making all antimicrobials available only by prescription. However, this is dependent on adequate and equitable access to veterinary expertise, alongside other regulatory measures to control importation, regulation and distribution of antimicrobials outside prescribing controls. In some countries, there may be access or cost barriers. Therefore, while curtailing over-the-counter access to antimicrobials is a worthy goal and is immediately feasible in many countries, it may have to be approached as a longer-term aspirational goal for regions that currently lack adequate expertise and resources. Increasing demand for animal protein in LMICs must be accompanied by improvements in animal health systems and will likely result in some undesirable but unavoidable AMU, as there will be some control measures that cannot be immediately implemented in some regions because of logistical, equity, and ethical reasons. While it is hoped that LMICs would not go through the same prolonged period of excessive AMU and substandard animal health systems that fostered the development of modern production systems in HICs, it must be understood that equitable access to meat protein and higher life standards means that it may be unfair to apply the same standards to developing regions with fewer resources, less developed infrastructure and fewer support mechanisms. The challenge for LMICs is to improve animal health systems in concert with increased food animal production, to minimize the need for the use of antimicrobials to compensate for sub-optimal animal management.

Elimination of antimicrobial growth promoters is a basic stewardship tool. Use of antimicrobials for growth promotion, a specific and definable situation, is amenable to reduction or elimination through international agreements and international standards, with parallel high-level efforts to advocate for and monitor implementation. Cessation of growth promotion would have no animal health or welfare consequences, but sudden and complete elimination may be challenging in regions that lack the ability to compensate through effective management improvements or financial supports. This should not be used as a reason to avoid restriction but recognizes that stepwise approaches may be required in some regions and/or the need for concurrent support mechanisms that can be included in pandemic instrument provisions that support equity. This can include phasing out use of growth promoters over time alongside provision of other supports, starting with immediate elimination of use of highest priority critically important antimicrobials for growth promotion,^12^ ultimately eliminating use of all medically important antimicrobials as growth promoters.

There may also be biological barriers to expected impacts of AMU restriction, including those that occur as a result of compensatory practices. For example, reduction of antimicrobial use for prevention of post-weaning diarrhea in pigs is sometimes compensated for by administration of high levels of dietary zinc, given the inhibitory effect that zinc has on *E. coli*. However, zinc can effectively select for AMR because zinc resistance genes can be co-located with AMR genes.^13^ There are also ecotoxicity concerns from feeding of zinc to livestock as this heavy metal is not metabolized or degraded, and will ultimately enter the environment. This demonstrates the potential unexpected consequences of AMU restriction and the need for post-intervention surveillance.

In most countries, there is a potential conflict of interest as veterinarians typically prescribe and dispense antimicrobials. This creates an apparent economic incentive to use antimicrobials, particularly more expensive drugs, which tend to be newer and broader spectrum. While it is unlikely that this is a driving factor for unnecessary AMU and there are no data suggesting that veterinarians are prescribing based on profit, this potential conflict of interest remains. A potential solution is removing the ability of veterinarians to dispense antimicrobials, but that would require marked changes in pharmacies and drug supply chains to ensure that animal owners had ready access to antimicrobials and that dispensing pharmacists have adequate knowledge of veterinary drugs. Controlling profit margins on antimicrobials sold by veterinarians could be considered as an alternative and has been successfully implemented in some European countries.^14^

While concerns about negative impacts of restriction typically focus on the economic impacts of animal disease or death, animal welfare cannot be ignored. While animal welfare cannot be used a reason to avoid restrictions, it must be considered as part of the complex and hard-to-quantify cost-benefit analyses. Similarly, the impact on the human-animal bond from illness or death of companion animals can be substantial at the individual level.

Ultimately, any improvements in animal health systems can underpin efforts to reduce AMU. Antimicrobials should not be used to compensate for poor animal management (e.g., poor biosecurity, inadequate nutrition, inadequate vaccination, excessive crowding, poor ventilation). Regulatory efforts need to consider improvements in animal health and welfare through improvements in animal health systems. Yet, it is challenging to implement international standards because of the wide range of production systems. There are also ethical challenges in requiring LMICs to adhere to standards developed for HICs, where there are better resources to implement improvements. Therefore, region- and sector- specific approaches are needed, ideally to provide a stepwise improvement in animal health systems in all areas, while not compromising food production, food security and economic viability of agriculture, particularly in LMICs.

## Designation of Antimicrobials for Human Use Only

One potential approach to reducing the impact of AMU in animals on AMR risks in humans is to limit the number of drug classes that are used in both sectors, through restriction of use in either food animals or animals as a whole. Restriction of antimicrobials that are commonly used in animals is challenging from animal health and welfare standpoints, but restriction of access to newer drugs that are not currently authorized in animals is more practical. This is particularly important for many newer human drugs that are often used as a last resort in critically ill patients where there are few or no other antimicrobial options. Need and cost often preclude use of these drugs in food animals and the main demand for use is likely for rare, sporadic use in individual companion animals, which may be justifiable, particularly with human-animal bond considerations. This again highlights the complexity of grouping all non-human animals together, as prohibiting use of new drug classes in food animals would be an easily justified approach, but facilitating exceptional use in companion animals may be reasonable and pose limited or negligible harm to humans when managed appropriately.

Access to multiple drug classes for animals is necessary because of the range of diseases that are encountered, so it can be challenging to effect significant changes in AMU through restriction of drug classes without potential impacts on animal health, animal welfare and food production. Restrictions in veterinary use of certain drug classes could also result in driving use underground if animal owners are able to obtain those drugs through other sources (e.g., internet, compounders, importation of active pharmaceutical ingredients). Limiting the number of drug classes available for use in animals would also concentrate a large volume of use of those classes, potentially increasing selection pressure for AMR for those classes and possibly other drug classes through cross-resistance or co-selection.

Conceptually, having separate and clearly distinct pools of antimicrobials for human and non-human use would be ideal. An ideal ‘animal only’ antimicrobial would be one that is safe, effective against pathogenic bacteria, low cost, and where there is no cross-resistance or co-selection with existing antimicrobials. However, that also describes an ideal human drug, so unless such a drug has those properties but can only be used in animals because of toxicity risks in humans, it would presumably be diverted for human use. This highlights the need for a pandemic instrument to have a focus on research and development, promoting national, regional and international activities to incentivize research and innovation activities that focus on alternatives to antimicrobials and improvements in health systems, versus development of new antimicrobials for the animal market.

## International Target Setting

Some ideas can be extrapolated from another insidious existential threat, climate change. A key aspect of the Paris Climate Agreement was recognition that international targets can be unifying and motivating.^15^ While it may be more challenging to develop internationally applicable targets for AMU and AMR, targets could facilitate national interest and momentum, and provide something more tangible for the public to understand and be motivated to help implement. Development and consistent use of international standards for measuring and reporting AMU is required, alongside assessment of optimal ways to report AMU for assessment of AMR risk. Wide variability between sectors and nations needs to be considered, but national and sector-specific monitoring and target development is a worthwhile goal and a data driven approach can provide more tangible outcomes to facilitate motivation, monitoring and broader acceptance of AMU optimization practices. Lack of regulatory framework was commonly cited as a barrier in AMR reporting in the 2021 WOAH (OIE) Annual Report on Antimicrobial Agents Intended for Use in Animals,^16^ something that must be addressed by individual countries. National and international efforts are required to improve AMU data collection and reporting, to facilitate accurate target setting. While participation in the voluntary WOAH report was strong (155/182 WOAH members), international agreements could widen participation.

## Regulatory vs. Individual Approaches

Measures to regulate AMU in animals can be implemented at a wide range of levels, including international, regional (e.g.,European Union) and national levels. There can also be sub-national efforts targeting specific commodity groups, voluntary restrictions by commodity groups, and efforts undertaken by individual animal owners, producers, veterinarians, feed mills, pharmaceutical companies, and consumers. For optimal control, a mix of interventions with involvement of everyone in the AMU ecosystem is required, from international bodies to individual antimicrobial users. However, there has been deferral to individuals or specific groups for antimicrobial stewardship initiatives, with fewer formal regulatory interventions. One can look again to climate change and the impacts that have been made on public awareness and acceptance. Initially, climate change strategies largely offloaded responsibility to individual sectors and downloaded responsibility from governments to individuals, something that is now recognized as being insufficient.^17^ A parallel with AMU can be considered, whereby most efforts are deferred to animal producers and antimicrobial prescribers, with less coordinated effort and support. This can be particularly problematic when there is limited motivation by users to effect change because problems and benefits are not readily apparent, and costs are born on the individual to largely produce benefits for society. Governmental and intergovernmental initiatives and instruments that focus efforts and responsibilities at higher government levels may help overcome some of these issues, and foster support and implementation of necessary adjunct components, such as improved surveillance, improved animal health systems, changes in antimicrobial access regulations and similar matters that would not be driven by efforts from individuals and organizations. An international agreement could drive the required social and economic commitments and transformations.

In parallel with regulatory practices, voluntary approaches can complement those efforts. Examples include voluntary industry-based restrictions (e.g., voluntary ban on the use of certain antimicrobials by a national commodity group), increased development and dissemination of evidence-based prescribing guidelines, animal health systems improvement support (especially in LMICs), AMU and AMR surveillance can be used to support and monitor these efforts.

## Conclusions

Antimicrobial resistance is a complex ecological problem that requires a complex ecological (One Health) solution involving a multitude of disciplines and sectors. There is not a single practical measure that would be expected to significantly impact AMR risks on its own. Therefore, broad efforts implementing and evaluating the use of multiple measures are required. Ultimately, the need for AMU is at the root of AMR and animal health systems underpin the need for AMU. Improvement in animal health systems including preventive measures and AMU practices can and must be achieved through various regulatory and non-regulatory approaches, to optimize AMU in animals, maintaining positive animal health and welfare impacts but minimizing the contribution to AMR in humans, animals and the environment.

## Note

This article reflects the views of Dr. Singh and should not be construed to represent FDA’s views or policies.

## References

[1] C.J.L. Murray , K.S. Ikuta , F. Sharara , et al., “Global Burden of Bacterial Antimicrobial Resistance in 2019: A Systematic Analysis,” The Lancet 399, no. 10325 (2022): 629–655. 10.1016/S0140-6736(21)02724-0 PMC8841637 35065702

[2] World Organization for Animal Health, Monitoring of the Quantities and Usage Patterns of Antimicrobial Agents Used in Food Producing Animals,” *Terrestrial Animal Health Code* (2022): at Chapter 6.9, *available at* <https://www.woah.org/fileadmin/Home/eng/Health_standards/tahc/current/chapitre_antibio_monitoring.pdf > (last visited October 4, 2022).

[3] S. Simjee , M. Henninger , G. Ippolito , and J. Atkinson , “Can We Align Antibiotic Policies at an International Level in the Absence of Harmonized Definitions?” Journal of Antimicrobial Chemotherapy 77, no. 3 (2022): 549–555. 35030247 10.1093/jac/dkab465

[4] *Id*.

[5] See *supra* note 2.

[6] World Health Organization, “Critically Important Antimicrobials for Human Medicine,” 6th rev. (2018), *available at* <https://www.who.int/publications-detail-redirect/9789241515528 > (last visited October 4, 2022).

[7] European Medicines Agency, C*ategorisation of Antibiotics for Use in Animals for Prudent and Responsible Use*, *available at* <https://www.ema.europa.eu/en/documents/report/infographic-categorisation-antibiotics-use-animals-prudent-responsible-use_en.pdf> (last visited October 4, 2022).

[8] World Health Organization, 2021 *AWaRe Classification* (2021), *available at* <https://www.who.int/publications/i/item/2021-aware-classification> (last visited October 4, 2022); World Health Organization, *WHO Model List of Essential Medicines - 22nd List, 2021*, (2021), *available at* <https://www.who.int/publications/i/item/WHO-MHP-HPS-EML-2021.02> (last visited October 4, 2022).

[9] World Organization for Animal Health, *OIE List of Antimicrobial Agents of Veterinary Importance*, 2018, *available at* <https://www.woah.org/app/uploads/2021/03/a-oie-list-antimicrobials-may2018.pdf> (last visited October 4, 2022).

[10] N. Dupont , L.H. Diness , M. Fertner , C.S. Kristensen , and H. Stege , “Antimicrobial Reduction Measures Applied in Danish Pig Herds Following the Introduction of the ‘Yellow Card’ Antimicrobial Scheme,” Preventive Veterinary Medicine 138 (2017): 9–16. 28237240 10.1016/j.prevetmed.2016.12.019

[11] European Union, *Regulation (EU)* 2019/6 *of the European Parliament and of the Council of 11 December 2018 on Veterinary Medicinal Products and Repealing Directive 2001/82/EC* (2019), *available at* <https://eur-lex.europa.eu/legal-content/EN/TXT/?uri=CELEX:32019R0006> (last visited October 4, 2022).

[12] *Supra* note 9.

[13] A. Moodley , S.S. Nielsen , and L. Guardabassi , “Effects of Tetracycline and Zinc on Selection of Methicillin-Resistant Staphylococcus Aureus (MRSA) Sequence Type 398 in Pigs,” *Veterinary Microbiol*ogy 152, nos. 3–4 (2011): 420–3. 10.1016/j.vetmic.2011.05.025 21664077

[14] L.P. Carmo , L.R. Nielsen , L. Alban , P.M. da Costa , G. Schupbach-Regula , and I. Magouras , “Veterinary Expert Opinion on Potential Drivers and Opportunities for Changing Antimicrobial Usage Practices in Livestock in Denmark, Portugal, and Switzerland,” Frontiers in Veterinary Science 5 (2018): 29. 29546044 10.3389/fvets.2018.00029 PMC5837977

[15] I. Weldon , S. Rogers Van Katwyk , G.L. Burci , et al.,” Governing Global Antimicrobial Resistance: 6 Key Lessons from the Paris Climate Agreement,” American Journal of Public Health 112, no. 4 (2022): 553–557. 35319963 10.2105/AJPH.2021.306695 PMC8961837

[16] World Organization for Animal Health, *OIE Annual Report on Antimicrobials Intended for Use in Animals* (2022), *available at* <https://www.woah.org/app/uploads/2021/05/a-fifth-annual-report-amr.pdf> (last visited October 4, 2022).

[17] See Weldon, et al., *supra* note 15.

